# Barrier-to-autointegration factor 1 promotes gammaherpesvirus reactivation from latency

**DOI:** 10.1038/s41467-023-35898-2

**Published:** 2023-02-06

**Authors:** Grant Broussard, Guoxin Ni, Zhigang Zhang, Qian Li, Patricio Cano, Dirk P. Dittmer, Blossom Damania

**Affiliations:** 1 Lineberger Comprehensive Cancer Center, University of North Carolina at Chapel Hill, Chapel Hill, NC 27599 USA; 2 Curriculum in Genetics and Molecular Biology, University of North Carolina at Chapel Hill, Chapel Hill, NC 27599 USA; 3 Department of Microbiology and Immunology, University of North Carolina at Chapel Hill, Chapel Hill, NC 27599 USA

**Keywords:** Herpes virus, Virus-host interactions, Pathogens, Viral infection

## Abstract

Gammaherpesviruses, including Kaposi sarcoma-associated herpesvirus (KSHV) and Epstein-Barr virus (EBV), are DNA viruses that are globally associated with human cancers and establish lifelong latency in the human population. Detection of gammaherpesviral infection by the cGAS-STING innate immune DNA-sensing pathway is critical for suppressing viral reactivation from latency, a process that promotes viral pathogenesis and transmission. We report that barrier-to-autointegration factor 1 (BAF)-mediated suppression of the cGAS-STING signaling pathway is necessary for reactivation of KSHV and EBV. We demonstrate a role for BAF in destabilizing cGAS expression and show that inhibiting BAF expression in latently infected, reactivating, or uninfected cells leads to increased type I interferon-mediated antiviral responses and decreased viral replication. Furthermore, BAF overexpression resulted in decreased cGAS expression at the protein level. These results establish BAF as a key regulator of the lifecycle of gammaherpesviruses and a potential target for treating viral infections and malignancies.

## Abstract (web summary)

Barrier-to-autointegration factor 1 (BAF) is a DNA binding protein involved in numerous pathways including transcriptional regulation, DNA damage response and nuclear assembly. Here, Broussard and colleagues characterise its role in gammaherpesvirus reactivation.

## Introduction

Gammaherpesviruses are widespread oncogenic human pathogens with significant disease burden. Kaposi sarcoma-associated herpesvirus (KSHV) causes human malignancies such as Kaposi sarcoma (KS), primary effusion lymphoma (PEL), and multicentric Castleman disease (MCD)^1–3^. Epstein–Barr virus (EBV) is the etiological agent of cancers such as nasopharyngeal carcinoma (NPC), gastric carcinoma, and various lymphomas^4^, as well as multiple sclerosis (MS)^5,6^. Gammaherpesviruses persist in a latent state of minimal gene expression with chromatin-tethered viral episomes replicating and dividing in synchrony with the cell. Latency is periodically disrupted by lytic replication, which involves broad viral gene expression, viral DNA amplification, and virion production^7,8^. Both lifecycle phases contribute to gammaherpesvirus persistence and disease pathogenesis^8,9^.

Lytic reactivation exposes gammaherpesviruses to detection by pattern recognition receptors (PRRs)^10,11^. Viral stimulation of PRRs activates innate immune signaling pathways that drive antiviral gene transcription, interferon production, or apoptosis^7,12^. Gammaherpesvirus infection and reactivation stimulates various PRRs, such as nucleotide-binding oligomerization domain (NOD)-like receptors (NLRs)^13,14^, retinoic acid-inducible gene I protein (RIG-I)-like receptors (RLRs)^15–19^, absent in melanoma 2 (AIM2)-like receptors (ALRs)^17,20–22^, Toll-like receptors (TLRs)^23–27^, interferon-gamma inducible protein 16 (IFI16)^20,28^, and cyclic GMP-AMP synthase (cGAS)^29^. Thus, evading these innate immune pathways is essential for gammaherpesvirus persistence and replication.

cGAS is an innate immune DNA sensor in the nuclear and cytoplasmic compartments^30,31^. After binding to dsDNA, cGAS catalyzes the synthesis of cyclic guanosine monophosphate-adenosine monophosphate (cGAMP), a second messenger molecule that activates stimulator of interferon genes (STING)^32^. STING induces TANK-binding kinase 1 (TBK1)-mediated phosphorylation of interferon regulatory factor 3 (IRF3), which promotes type I interferon gene transcription^33^. KSHV and EBV encode factors which target the cGAS-STING DNA-sensing pathway. KSHV ORF52 (KicGAS) and its EBV homolog, BLRF2, block cGAMP production by binding cGAS and inhibiting enzymatic activity^34^. KSHV latency-associated nuclear antigen (LANA) also binds cGAS, preventing downstream TBK1 phosphorylation and activation^35^. KSHV viral interferon regulatory factor 1 (vIRF1) binds STING to prevent TBK1 recruitment^29^. Gammaherpesviruses also co-opt host factors to subvert cGAS-STING signaling. KSHV recruits protein phosphatase Mg^2+^/Mn^2+^ dependent 1G (PPM1G) to STING, shutting down cGAS-STING signaling^36^. EBV infection upregulates tripartite motif-containing protein 29 (TRIM29), which ubiquitinates and degrades STING, dampening innate immunity^37^.

To prevent autoimmunity, cells encode factors that regulate cGAS-STING signaling. Barrier-to-autointegration factor 1 (BAF), encoded by *BANF1*, is a singular DNA-binding protein (distinct from the chromatin remodeling SWI/SNF complex of the same name) with various functions, including regulating nuclear assembly, DNA damage responses, and recruitment of transcription factors or epigenetic modifiers^38–40^. BAF competes with cGAS for access to DNA in the nucleus and the cytoplasm, thereby preventing sensing of host DNA^41^. Since cGAS-STING signaling is critical for detecting gammaherpesviruses^29,34,35,37,42,43^, we investigated the role of BAF in the lifecycle of KSHV and EBV. We demonstrate that BAF plays a pro-viral role in gammaherpesviral reactivation from latency. BAF depletion in KSHV-infected cells led to impaired lytic gene transcription during reactivation and defects in the production of lytic proteins and infectious virions. BAF depletion during reactivation increased type I interferon responses in a cGAS-STING-IRF3-dependent manner. BAF overexpression rendered cells more permissive to KSHV lytic reactivation. In addition, BAF knockdown limited lytic reactivation of a related herpesvirus, EBV, through a similar mechanism, suggesting that BAF-mediated innate immunosuppression facilitates the gammaherpesvirus lifecycle.

## Results

### BAF is required for optimal KSHV reactivation from latency

**Fig. 1:**
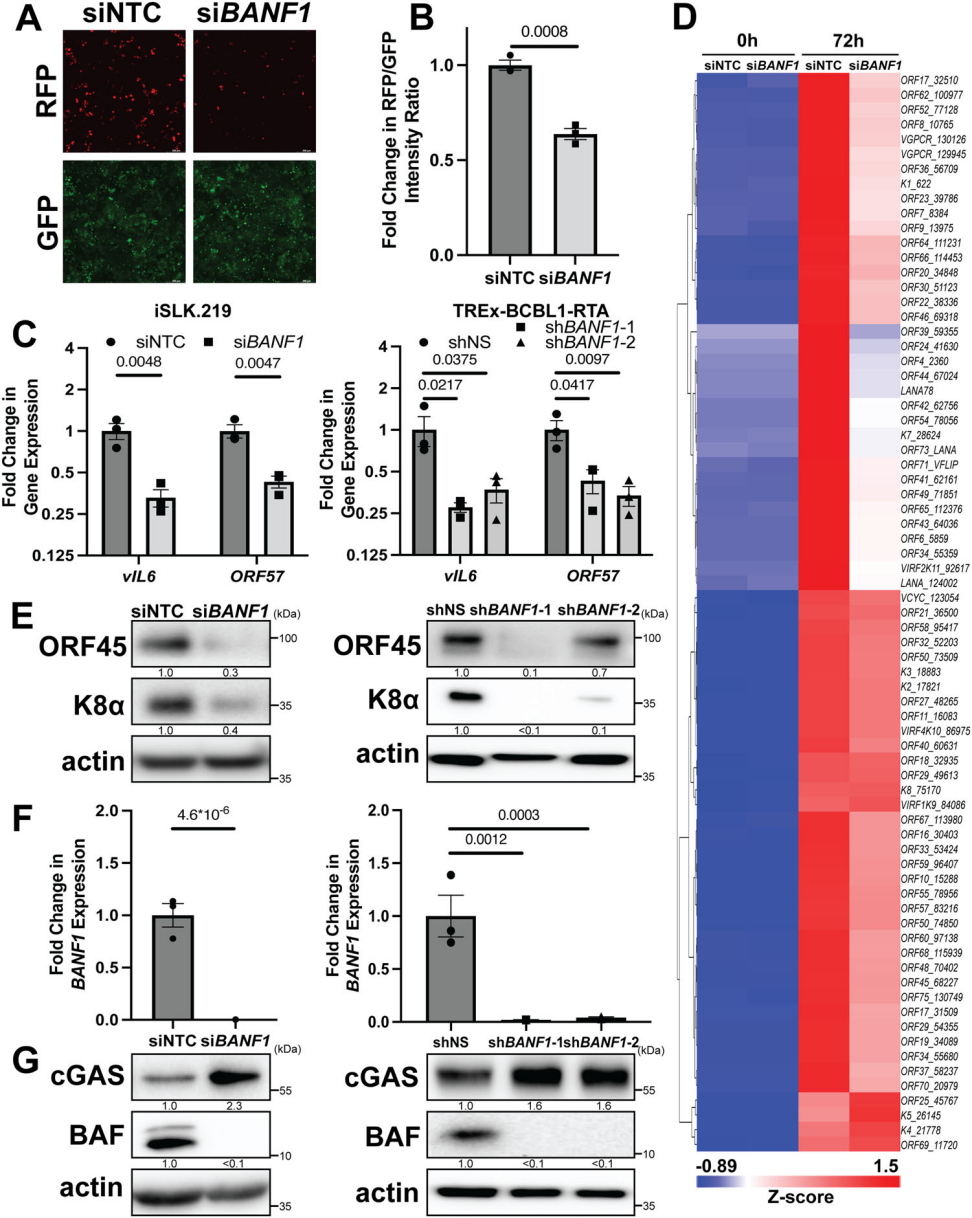
BAF is required for optimal KSHV reactivation from latency. iSLK.219 cells were transfected with non-targeting control (NTC) siRNA or *BANF1* targeting siRNA 48 h prior to the addition of 25 ng/mL doxycycline. TREx-BCBL1-RTA cells were transduced with lentiviral shRNA for 72 h prior to the addition of 1000 ng/mL doxycycline. **A** Fluorescent microscopy imaging of iSLK.219 cells for RFP and GFP signal at 72 h post-doxycycline treatment. **B** The fluorescence was quantified by plate reader. **C** Cells were harvested for RNA at 72 h (iSLK.219) or 96 h (TREx-BCBL1-RTA) post-doxycycline treatment and subsequent RT-qPCR was performed to quantify expression of viral mRNA transcripts. **D** iSLK.219 cDNA was prepared from cells harvested at 0 h and 72 h post-doxycycline treatment. Global KSHV gene expression profiling was performed. Data shown are the Z-score of the fold change (2^-ΔΔCt^) over the geometric mean expression of three housekeeping genes averaged over two independent biological replicates. The heatmap was prepared using Partek Flow. **E** Cell lysates were prepared at 72 h (iSLK.219) or 96 h (TREx-BCBL1-RTA) post-doxycycline treatment and analyzed by western blotting with the indicated antibodies. **F** Cells were harvested and RNA isolated at 48 h post-siRNA transfection and RT-qPCR was subsequently performed to quantify *BANF1* mRNA transcripts. **G** Cell lysates were prepared at 48 h post-siRNA transfection or 72 h post-shRNA transduction and analyzed by western blotting with the indicated antibody. *P* values are the result of two-tailed Student’s *T* tests unless otherwise specified. Error bars indicate the standard error of the mean of three independent biological replicates. Source data are provided as a source data file.

To investigate a potential role for BAF in KSHV lytic reactivation, we employed the iSLK.219 cell line, which is latently infected with a recombinant KSHV virus (rKSHV.219) that constitutively expresses GFP and conditionally expresses RFP upon lytic reactivation using the KSHV polyadenylated nuclear RNA (*PAN*) promoter^44^. As an orthogonal method, we employed the TREx-BCBL1-RTA cell line, a KSHV-infected primary effusion lymphoma-derived B-cell line^45^. Both cell lines express key viral lytic transactivator KSHV replication and transcription activator (RTA) using a doxycycline-inducible promoter. Hence, although cells contain latent KSHV, viral reactivation can be induced by doxycycline treatment. We transfected iSLK.219 cells with siRNA targeting *BANF1* transcripts or non-targeting control (NTC) siRNA and 48 h later induced them to reactivate the virus by treating them with doxycycline. At 72 h post-induction, we detected significantly less RFP signal in BAF-depleted cells compared to the control, suggesting a defect in reactivation (Fig. 1A, B). Transfection with *BANF1* siRNA rather than NTC siRNA led to defects in the expression of lytic KSHV transcripts, such as *vIL6* and *ORF57*, in iSLK.219 cells and in TREx-BCBL1-RTA cells (Fig. 1C and Supplementary Fig. 1A). BAF was depleted in TREx-BCBL1-RTA cells via infection with lentiviruses encoding two independent shRNAs targeting *BANF1* transcripts prior to reactivation. This also resulted in significantly decreased lytic KSHV mRNA production compared to cells infected with non-specific (NS) shRNA lentivirus (Fig. 1C).

A KSHV genome RT-qPCR profiling array revealed impaired expression of most viral transcripts at 72 h post-reactivation in BAF-depleted iSLK.219 cells (Fig. 1D). Reduced KSHV lytic transcript expression correlated with reduced expression of KSHV lytic proteins, such as ORF45 and K8α, in BAF-depleted iSLK.219 and TREx-BCBL1-RTA cells compared to control cells. A similar phenotype was observed with lentiviral BAF shRNA knockdown in TREx-BCBL1-RTA cells (Fig. 1E and Supplementary Fig. 1B). BAF knockdown by siRNA treatment or lentiviral shRNA transduction was confirmed by RT-qPCR for *BANF1* mRNA transcripts and western blotting for BAF protein (Fig. 1F, G and Supplementary Fig. 1C, D). Strikingly, BAF knockdown correlated with increased cGAS protein levels in KSHV-infected cells (Fig. 1G and Supplementary Fig. 1D). These data suggest a role for BAF in facilitating optimal KSHV reactivation that involves the cGAS-STING pathway.

### BAF antagonizes the cGAS-STING response to KSHV reactivation

**Fig. 2:**
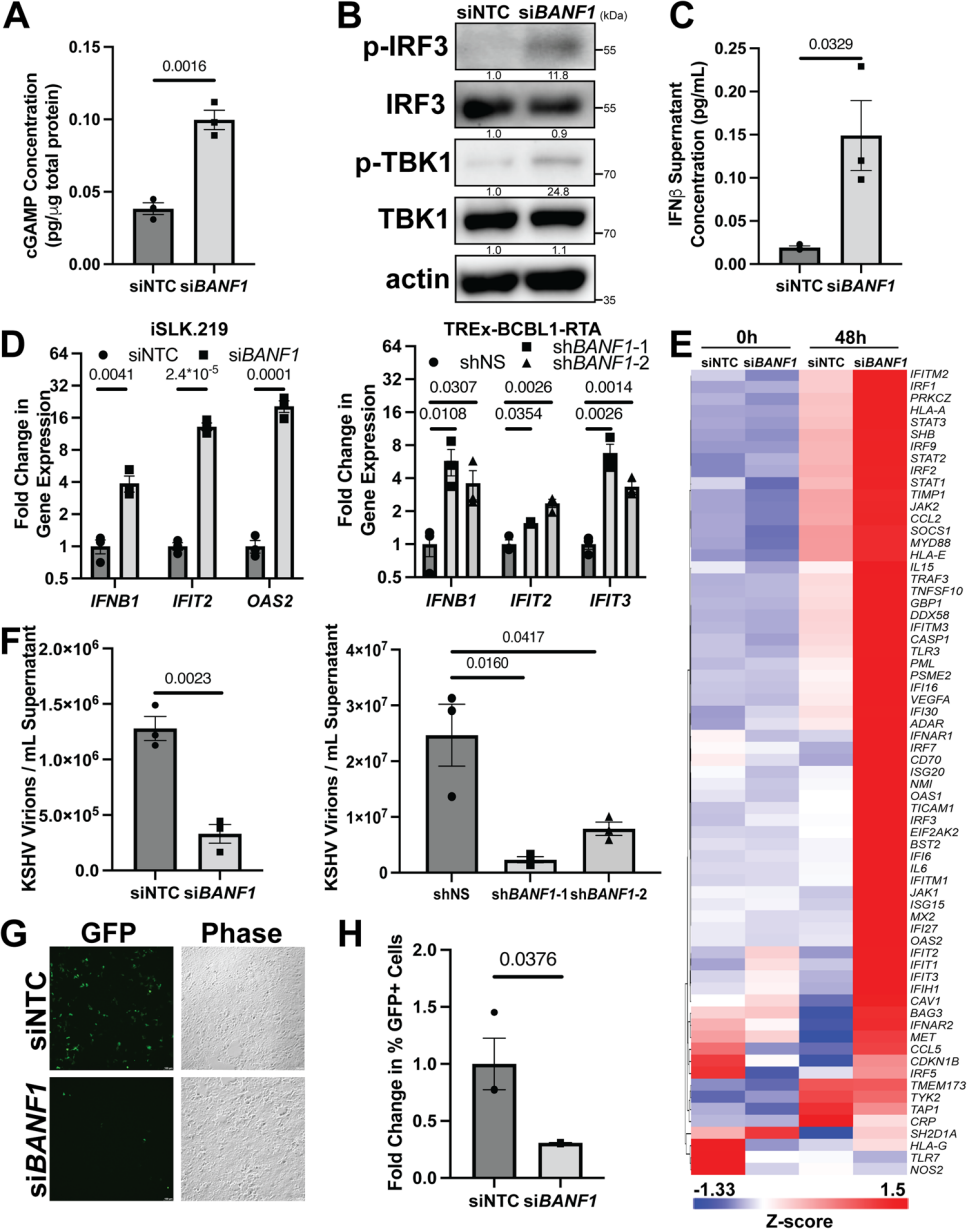
BAF antagonizes the cGAS-STING response to KSHV reactivation. iSLK.219 cells were transfected with NTC siRNA or *BANF1* targeting siRNA for 48 h prior to the addition of 25 ng/mL doxycycline. TREx-BCBL1-RTA cells were transduced with lentiviral shRNA for 72 h prior to the addition of 1000 ng/mL doxycycline. **A** iSLK.219 cell lysates were collected at 72 h post-doxycycline treatment and analyzed by 2’3’-cGAMP ELISA. **B** iSLK.219 cell lysates were collected at 48 h post-doxycycline treatment and analyzed by western blotting with the indicated antibodies. **C** iSLK.219 culture supernatant was harvested at 72 h post-doxycycline treatment and analyzed by IFNβ ELISA. **D** Cells were harvested for RNA at 48 h (iSLK.219) or 96 h (TREx-BCBL1-RTA) post-doxycycline treatment and RT-qPCR was subsequently performed to determine ISG mRNA expression levels. **E** iSLK.219 cDNA as prepared in (**D**) was analyzed by Human Type I Interferon Response RT^2^ Profiler PCR Array (Qiagen, GeneGlobe ID: PAHS-016Z). Data shown are the Z-score of the fold change (2^-ΔΔCt^) over the geometric mean expression of four housekeeping genes averaged over two independent biological replicates. The heatmap was prepared using Partek Flow. **F** Culture supernatants were harvested at either 72 h (iSLK.219) or 96 h (TREx-BCBL1-RTA) post-doxycycline treatment and DNase treated prior to DNA extraction. DNase-resistant KSHV genomes were quantified by real-time qPCR. **G** iSLK.219 culture supernatants were transferred to naive HEK293 cells at 72 h post-doxycycline treatment. GFP + infected cells were measured at 48 h post-infection by fluorescent microscopy and (**H**) quantified by flow cytometry. *P* values are the result of two-tailed Student’s *T* tests unless otherwise specified. Error bars indicate the standard error of the mean of three independent biological replicates. Source data are provided as a source data file.

Since BAF knockdown restricted KSHV reactivation and elevated cGAS protein expression, we investigated the activation of the entire cGAS-STING-IRF3 pathway. *BANF1* siRNA transfected iSLK.219 cells produced more cGAMP, the enzymatic product of cGAS, than NTC siRNA-treated cells during KSHV reactivation (Fig. 2A). Increased cGAMP production correlated with increased TBK1 and IRF3 phosphorylation, evidencing activation of downstream effectors of cGAS (Fig. 2B). Increased pathway activation resulted in increased secretion of IFNβ protein, a type I interferon, into the medium of reactivating cells with reduced BAF expression (Fig. 2C). This, in turn, increased expression of ISGs, such as genes encoding interferon-induced protein with tetratricopeptide repeats (IFIT) proteins, oligoadenylate synthase (OAS) proteins, and IFNβ protein in iSLK.219 and TREx-BCBL1-RTA cells treated with siRNA targeting *BANF1* (Fig. 2D and Supplementary Fig. 2A). Lentiviral BAF shRNA transduction of TREx-BCBL1-RTA cells prior to lytic reactivation similarly increased ISG expression during reactivation compared to cells containing control shRNA (Fig. 2D). We also profiled interferon responses in BAF-depleted cells using an RT-qPCR array (Qiagen, GeneGlobe ID: PAHS-016Z). The type I interferon response to KSHV reactivation was broadly augmented when BAF expression was suppressed by siRNA treatment in iSLK.219 cells (Fig. 2E). These results demonstrate that BAF antagonizes the cGAS-STING response to KSHV reactivation.

Augmented antiviral responses corresponded with a reduction in encapsidated KSHV genomes in BAF-depleted iSLK.219 or TREx-BCBL1-RTA cell supernatants compared to control cell supernatants (Fig. 2F and Supplementary Fig. 2B), indicating inhibition of virion assembly and maturation. Direct comparison of variables measured in different cell types, such as viral titer, are graphically represented on a common axis in Supplementary Fig. 8. Similarly, lentiviral transduction with shRNAs targeting *BANF1* transcripts prior to reactivation resulted in significantly decreased encapsidated KSHV genome production compared to cells infected with non-specific shRNA lentivirus (Fig. 2F). To extend these results, we measured changes in infectious particles. Supernatant transferred from reactivated BAF-depleted iSLK.219 cells displayed significantly less KSHV infection of naive HEK293 cells as assayed by GFP positivity than supernatant transferred from reactivated NTC siRNA transfected cells (Fig. 2G, H and Supplementary Fig. 2C). These data demonstrate that the pro-viral role of BAF in KSHV reactivation involves downregulating cGAS-STING innate immune responses.

### Increased BAF expression promotes KSHV reactivation

**Fig. 3:**
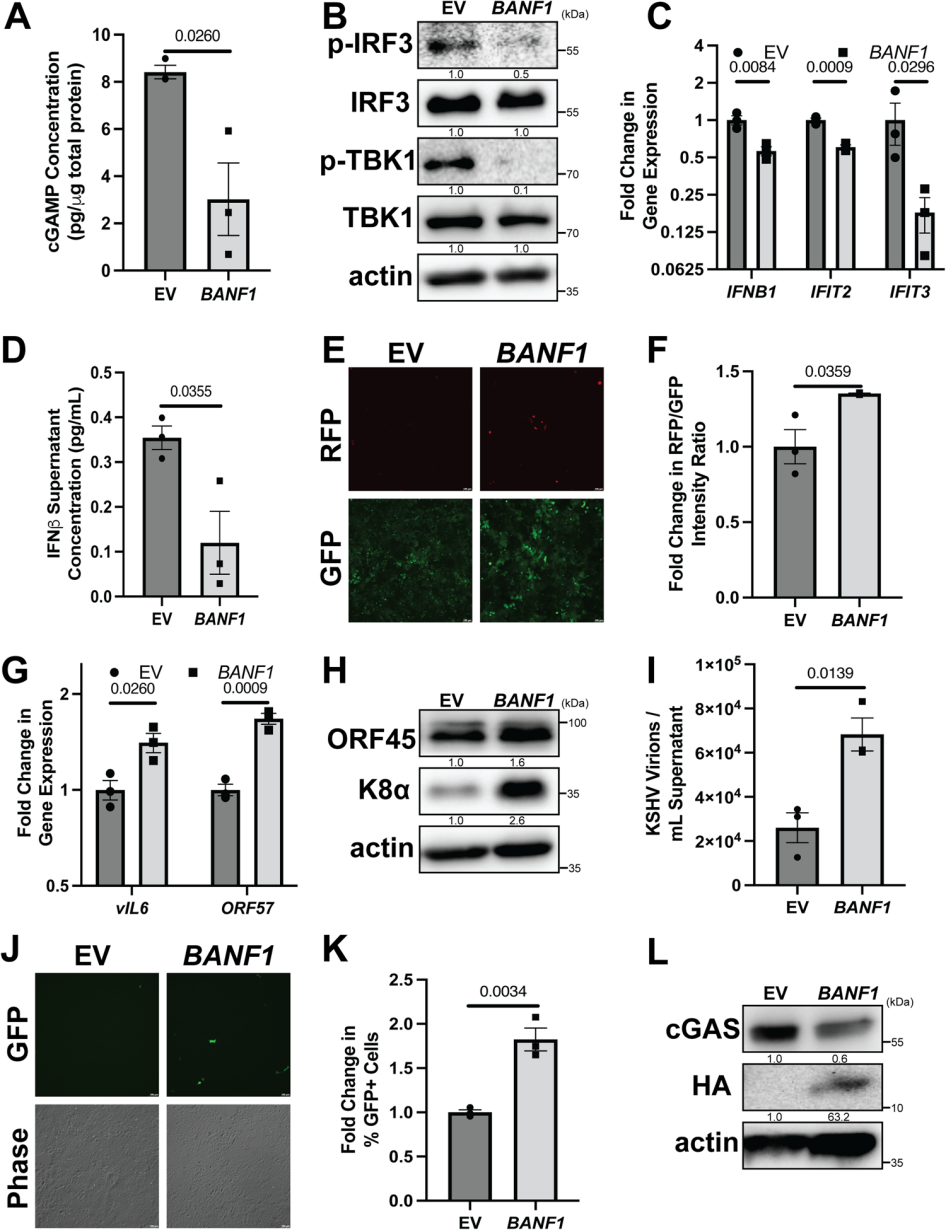
Increased BAF expression promotes KSHV reactivation. iSLK.219 cells were transfected with either pCMV6 (EV) or pCMV6-*BANF1*-HA (BAF) expression plasmid for 48 h prior to the addition of 25 ng/mL doxycycline. **A** Cell lysates were collected at 96 h post-doxycycline treatment and analyzed by 2’3’-cGAMP ELISA. **B** Cell lysates were collected at 72 h post-doxycycline treatment and analyzed by western blotting with the indicated antibodies. **C** Cells were harvested and RNA was isolated at 72 h post-doxycycline treatment and subsequent RT-qPCR was performed to determine ISG mRNA expression levels. **D** Culture supernatant was harvested at 96 h post-doxycycline treatment and analyzed by IFNβ ELISA. **E** Fluorescent microscopy imaging of RFP and GFP signal was conducted at 96 h after doxycycline treatment and quantified (**F**) by plate reader. **G** Cells were harvested for RNA at 96 h post-doxycycline treatment and subsequent RT-qPCR was performed to quantify the expression of viral mRNA transcripts. **H** Cell lysates were prepared at 96 h post-doxycycline treatment and analyzed by western blotting with the indicated antibodies. **I** Culture supernatants were harvested at 96 h post-doxycycline treatment and DNase treated prior to DNA extraction. DNase-resistant KSHV genomes were quantified by real-time qPCR. **J** At 96 h post-doxycycline treatment, culture supernatants from iSLK.219 cells were used to infect naive HEK293 cells. GFP + infected cells were measured at 48 h post-transfer by fluorescent microscopy and (**K**) quantified by flow cytometry. **L** Cell lysates were prepared at 48 h post-siRNA transfection and analyzed by western blotting with the indicated antibody. *P* values are the result of two-tailed Student’s *T* tests unless otherwise specified. Error bars indicate the standard error of the mean of three independent biological replicates. Source data are provided as a source data file.

To independently confirm the role of BAF in viral reactivation, we overexpressed BAF using an HA-tagged *BANF1* expression plasmid. Transfecting iSLK.219 cells with the BAF-expressing plasmid prior to KSHV lytic reactivation led to decreased cGAMP production compared to cells transfected with empty vector (EV) (Fig. 3A). Phosphorylation levels of cGAS-STING pathway components, TBK1 and IRF3, were reduced in cells overexpressing BAF, indicating a decrease in signaling (Fig. 3B). Suppression of this pathway correlated with a decrease in ISG mRNA expression, including *IFNB1*, and reduced amounts of IFNβ in cell supernatants (Fig. 3C, D). Decreased antiviral signaling in BAF-overexpressing iSLK.219 cells resulted in increased KSHV transcriptional activity, as assayed by RFP reporter signal and RT-qPCR for signature KSHV lytic mRNA transcripts such as *vIL6* and *ORF57* (Fig. 3E–G). KSHV lytic proteins, such as ORF45 and K8α, were increased in iSLK.219 cells consequent to BAF overexpression (Fig. 3H), and supernatants from iSLK.219 cells transfected with a BAF-expressing plasmid contained more encapsidated KSHV genomes and displayed greater infectivity upon transfer to naive HEK293 cells than supernatants from cells transfected with an empty vector control plasmid (Fig. 3I–K and Supplementary Fig. 3). Strikingly, increased BAF expression correlated with a reduction in expression of cGAS protein (Fig. 3L). These data confirm that BAF plays a pro-viral role in KSHV reactivation by suppressing the cGAS-STING innate immune response.

### BAF promotes KSHV reactivation through a cGAS-dependent mechanism

**Fig. 4:**
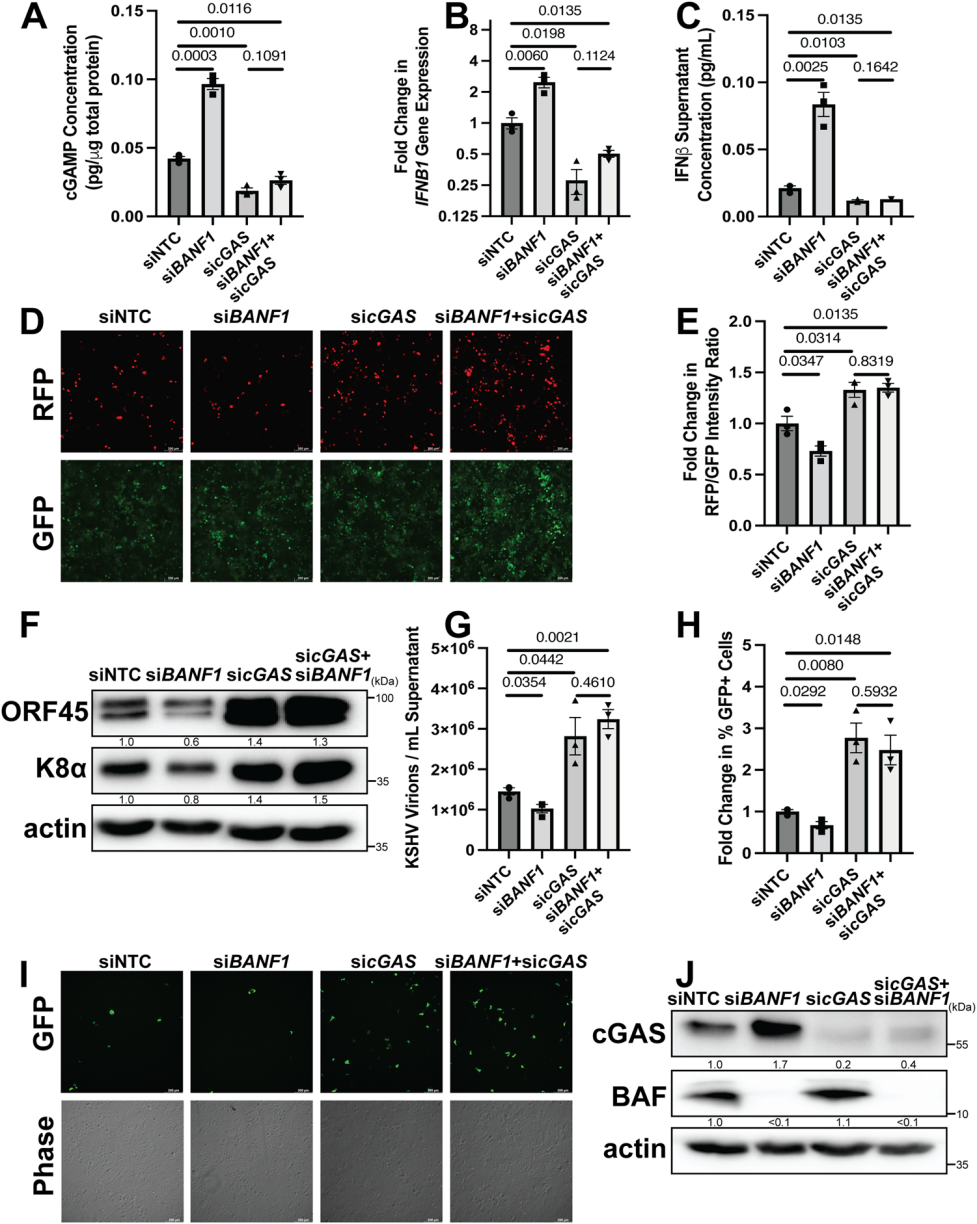
BAF promotes KSHV reactivation through a cGAS-dependent mechanism. iSLK.219 cells were transfected with NTC, *BANF1*, *cGAS*, or *BANF1* and *cGAS* targeting siRNA at a total siRNA concentration of 100 nM for 48 h prior to the addition of 25 ng/mL doxycycline. **A** Cell lysates were collected at 72 h post-doxycycline treatment and analyzed by 2’3’-cGAMP ELISA. **B** Cells were harvested for RNA at 48 h post-doxycycline treatment and subsequent RT-qPCR was performed to determine *IFNB1* mRNA expression levels. **C** Culture supernatant was harvested at 72 h post-doxycycline treatment and analyzed by IFNβ ELISA. **D** Fluorescent microscopy imaging of RFP and GFP signal was conducted at 72 h after doxycycline treatment. **E** The fluorescence was quantified by a plate reader. **F** Cell lysates were prepared at 72 h post-doxycycline treatment and analyzed by western blotting with the indicated antibodies. **G** Culture supernatants were harvested 72 h post-doxycycline treatment and DNase treated prior to DNA extraction. DNase-resistant KSHV genomes were quantified by real-time qPCR. **H** At 72 h post-doxycycline treatment, culture supernatants from iSLK.219 cells were used to infect naive HEK293 cells. At 48 h post-transfer, GFP + infected cells were quantified by flow cytometry. **I** The cells were also analyzed by fluorescent microscopy. **J** Cell lysates were prepared at 48 h post-siRNA transfection and analyzed by western blotting with the indicated antibody. *P* values are the result of two-tailed Student’s *T* tests unless otherwise specified. Error bars indicate the standard error of the mean of three independent biological replicates. Source data are provided as a source data file.

To ensure that the suppression of antiviral signaling by BAF was specifically mediated through the cGAS-STING-IRF3 signaling axis, we conducted BAF knockdown experiments in iSLK.219 cells either with or without simultaneous cGAS knockdown. Transfection of iSLK.219 cells with siRNA targeting *cGAS* facilitates KSHV lytic reactivation^29^. Consistent with these prior findings, cGAS depletion led to significantly less cGAMP production in reactivating iSLK.219 cells but was not significantly increased by simultaneous BAF knockdown (Fig. 4A). Likewise, *IFNB1* mRNA levels in cells transfected with *cGAS* siRNA were consistently lower and unaffected by co-transfection with *BANF1* siRNA (Fig. 4B). This was mirrored by IFNβ protein levels observed in the supernatant of reactivating iSLK.219 cells, where there was no significant difference between transfection with *cGAS* siRNA alone or with a combination of *cGAS* and *BANF1* siRNA (Fig. 4C). These results support the model that cGAS is epistatic to BAF, i.e., both operate in the same pathway with respect to KSHV reactivation.

KSHV lytic transcriptional activity in iSLK.219 cells, as measured by *PAN* promoter-dependent RFP signal and levels of KSHV lytic mRNA transcripts (*vIL6* and *ORF57*), was uniformly higher when cGAS was knocked down whether or not BAF was simultaneously knocked down (Fig. 4D, E and Supplementary Fig. 4A). BAF depletion reduced KSHV lytic protein levels in reactivating iSLK.219 cells, but lytic protein expression was uniformly higher when cGAS was depleted whether or not BAF was simultaneously depleted (Fig. 4F). Levels of encapsidated virions and infectious titer were reduced in BAF-depleted cells compared to control cells and were increased in cGAS-depleted cells compared to control cells. When BAF and cGAS were both depleted, the results mirrored the cGAS depletion phenotype. (Fig. 4H, I and Supplementary Fig. 4B). Downregulation of BAF, cGAS, or both was verified by western blotting and RT-qPCR for *BANF1* and *cGAS* mRNA transcript levels (Fig. 4J and Supplementary Fig. 4C). These data confirm that the pro-viral role BAF plays in KSHV reactivation is cGAS-dependent.

### BAF suppresses the antiviral state in uninfected, latent, and lytic cells by altering cGAS stability

**Fig. 5:**
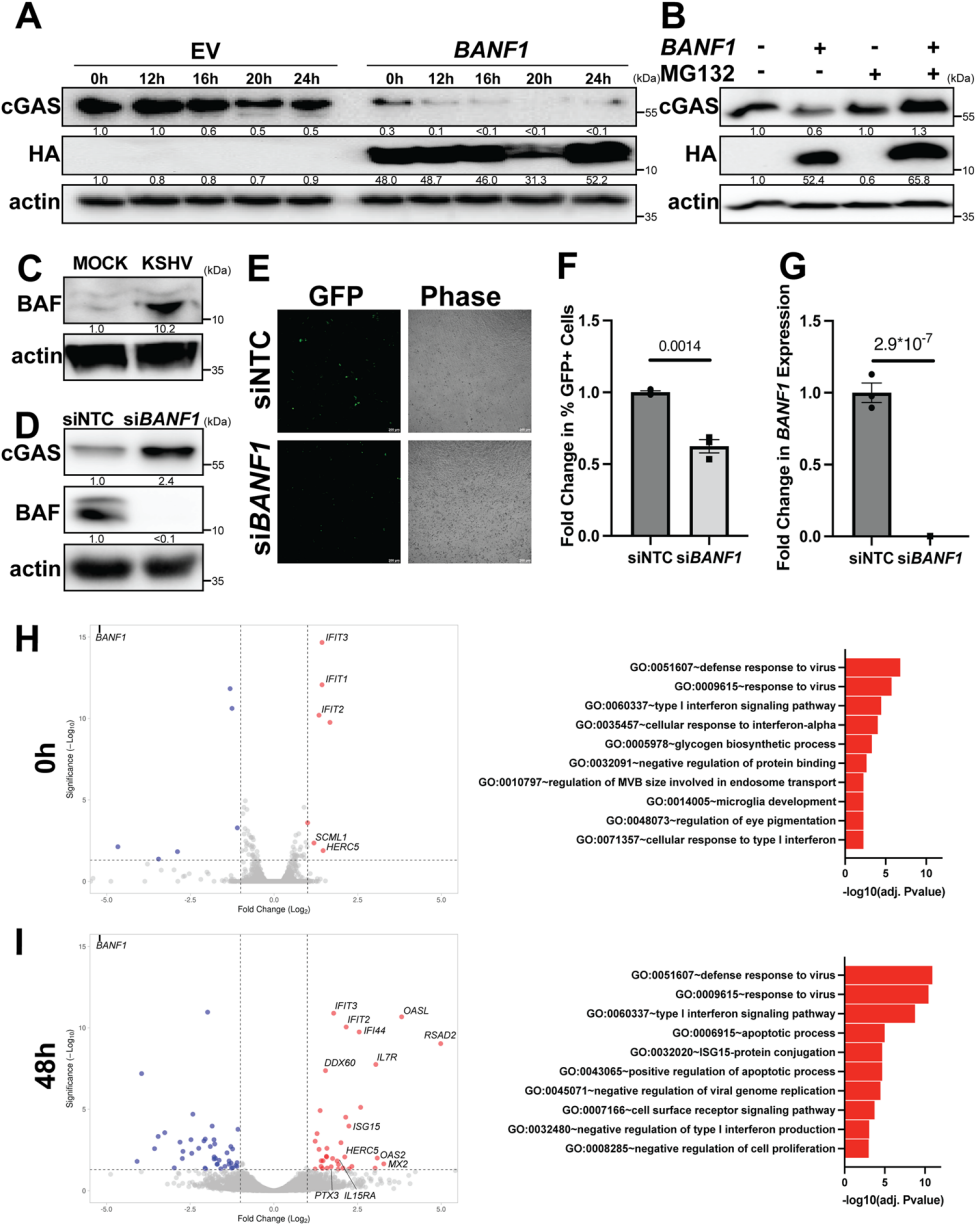
BAF suppresses the antiviral state in uninfected, latently infected, and lytically infected cells. **A** iSLK.219 cells were transfected with EV or *BANF1* expression plasmids for 48 h prior to the addition of 100 μg/mL cycloheximide. Cell lysates were prepared at the indicated timepoints and analyzed by western blotting. **B** HeLa cells were transfected with EV or *BANF1* expression plasmids for 48 h prior to treatment with 10 μM MG132 or DMSO control. Cell lysates were prepared 12 h post-treatment and analyzed by western blotting. **C** Naive SLK cells were infected with equivalent units of concentrated cell-free KSHV or PBS (mock). Cell lysates were prepared 6 h post-infection and analyzed by western blotting. **D** Naive SLK cells were transfected with *BANF1* or NTC siRNA for 48 h prior to infection with equivalent units of concentrated cell-free KSHV. Lysates were prepared pre-infection and analyzed by western blot. Blots are representative of three independent biological replicates. **E** GFP + infected cells were analyzed at 48 h post-infection by fluorescent microscopy. **F** Cells were also quantified by flow cytometry. **G** Cells were harvested and RNA isolated at 48 h post-siRNA transfection and RT-qPCR was subsequently performed to quantify *BANF1* mRNA transcripts. iSLK.219 cells were transfected with NTC siRNA or *BANF1* targeting siRNA for 48 h prior to the addition of 25 ng/mL doxycycline. Cells from two biological replicates were harvested for RNA (**H**) at 0 h and (**I**) at 48 h post-doxycycline treatment and subjected to RNA-Seq analysis. Interferon-stimulated genes were identified using Interferome (http://www.interferome.org)^70^. Data were visualized with VolcaNoseR (https://huygens.science.uva.nl/VolcaNoseR/)^69^. Adjusted *P* values were calculated using the two-tailed Wald test with adjustments for multiple comparisons, and significantly enriched GO terms were determined by the one-tailed Fisher exact test with adjustments for multiple comparisons. *P* values are the result of two-tailed Student’s *T* tests unless otherwise specified. Error bars indicate the standard error of the mean of three independent biological replicates. Source data are provided as a source data file.

We next sought to investigate the mechanism by which cGAS protein levels are regulated by BAF expression in iSLK.219 cells. BAF overexpression prior to treatment with cycloheximide, a protein synthesis inhibitor, caused existing cellular cGAS pools to decay at an increased rate compared to EV transfected cells (Fig. 5A and Supplementary Fig. 5B), while *BANF1* siRNA treatment had the opposite effect when compared to NTC (Supplementary Fig. 5A, C). To determine the mechanism by which BAF induces the degradation of cGAS, we overexpressed BAF in HeLa cells treated with either MG132, a proteasome inhibitor, or DMSO control. While BAF overexpression induced degradation of cGAS in the DMSO control cells, it had no such effect in the context of MG132 treatment, suggesting that BAF-mediated cGAS degradation requires the proteasome (Fig. 5B). Direct binding of myc-tagged cGAS to HA-tagged BAF was not observed via immunoprecipitation assays performed under stringent conditions with RIPA buffer (Supplementary Fig. 5D). When immunoprecipitations were performed under less stringent conditions using NP40 buffer, an interaction was observed between myc-tagged cGAS and HA-tagged BAF. However, treatment of the lysate with DNase prior to immunoprecipitation abolished this interaction, suggesting that the complex observed was indirect, mediated by mutual DNA binding (Supplementary Fig. 5E). These results are consistent with the literature demonstrating that BAF and cGAS both bind DNA^41^. These data confirm that BAF expression contributes to cGAS degradation via a proteasomal mechanism in the unprimed, equilibrium state.

We hypothesized that BAF-mediated cGAS degradation may potentially be co-opted by the virus for immune evasion during primary infection to establish latency. We infected uninfected SLK cells with rKSHV.219 virus. Six hours post-infection with cell-free rKSHV.219, BAF protein levels were increased compared to mock infection in SLK cells (Fig. 5C). Efficient infection of the SLK cell population was confirmed by fluorescent microscopy for GFP expression and western blotting for LANA expression (Supplementary Fig. 5F, G).

Since BAF contributed to cGAS degradation and was upregulated early in primary infection, we hypothesized that a BAF knockdown may also protect naive cells from subsequent primary infection with KSHV. When SLK cells were treated with *BANF1* or NTC siRNA prior to infection with rKSHV.219, the cGAS expression of *BANF1* siRNA transfected cells was increased compared to NTC (Fig. 5D). This correlated with a smaller percentage of BAF-depleted cells becoming infected as assayed by GFP positivity compared to control cells (Fig. 5E, F and Supplementary Fig. 5H). BAF depletion was confirmed by western blotting and RT-qPCR for *BANF1* mRNA transcripts (Fig. 5D, G).

To further characterize the BAF-cGAS-KSHV interplay, we compared RNA-Seq profiles of *BANF1* or NTC siRNA-treated iSLK.219 cells before and after KSHV reactivation (0 h and 48 h post-doxycycline induction). Prior to lytic reactivation (0 h), BAF-depleted cells already exhibited a stronger antiviral state, with the top three significantly enriched gene ontology (GO) analysis terms relating to antiviral responses (Fig. 5H). Five of the seven genes that were significantly upregulated greater than twofold were ISGs (Supplementary Data 1). IFIT proteins play a role in the type I interferon response to KSHV infection, while HECT and RLD domain containing E3 ubiquitin protein ligase 5 (HERC5) is the E3 ubiquitin ligase for ISG15 ubiquitin-like modifier (ISG15), an antiviral post-translational modification known to target KSHV proteins^46,47^. The reduced expression of some genes in BAF-depleted cells, e.g., RNA Polymerase III Subunit A (*POLR3A*), was associated with induction of host defense responses to viruses, while many of the other downregulated genes corresponded to GO terms unrelated to the pro-viral phenotype. At 48 h after lytic induction, differences in antiviral gene expression between *BANF1* or NTC siRNA transfected iSLK.219 cells were even larger than those observed in latency (Fig. 5I). Thirty-two of the thirty-six genes that were significantly upregulated greater than twofold were ISGs (Supplementary Data 1). Genes encoding known KSHV antagonists such as OAS proteins, interferon-induced protein 44 (IFI44), and DExD/H-Box helicase 60 (DDX60) were upregulated in lytically reactivating BAF-depleted cells, in addition to IFIT- and ISG15-related transcripts that were also upregulated in latent BAF-depleted cells^46,48^. These data underscore that BAF plays a role in suppressing cGAS-STING innate immune defenses in all stages of viral infection: in uninfected cells promoting primary infection, in latently infected cells, and in lytically reactivating cells.

### BAF facilitates EBV reactivation from latency

**Fig. 6:**
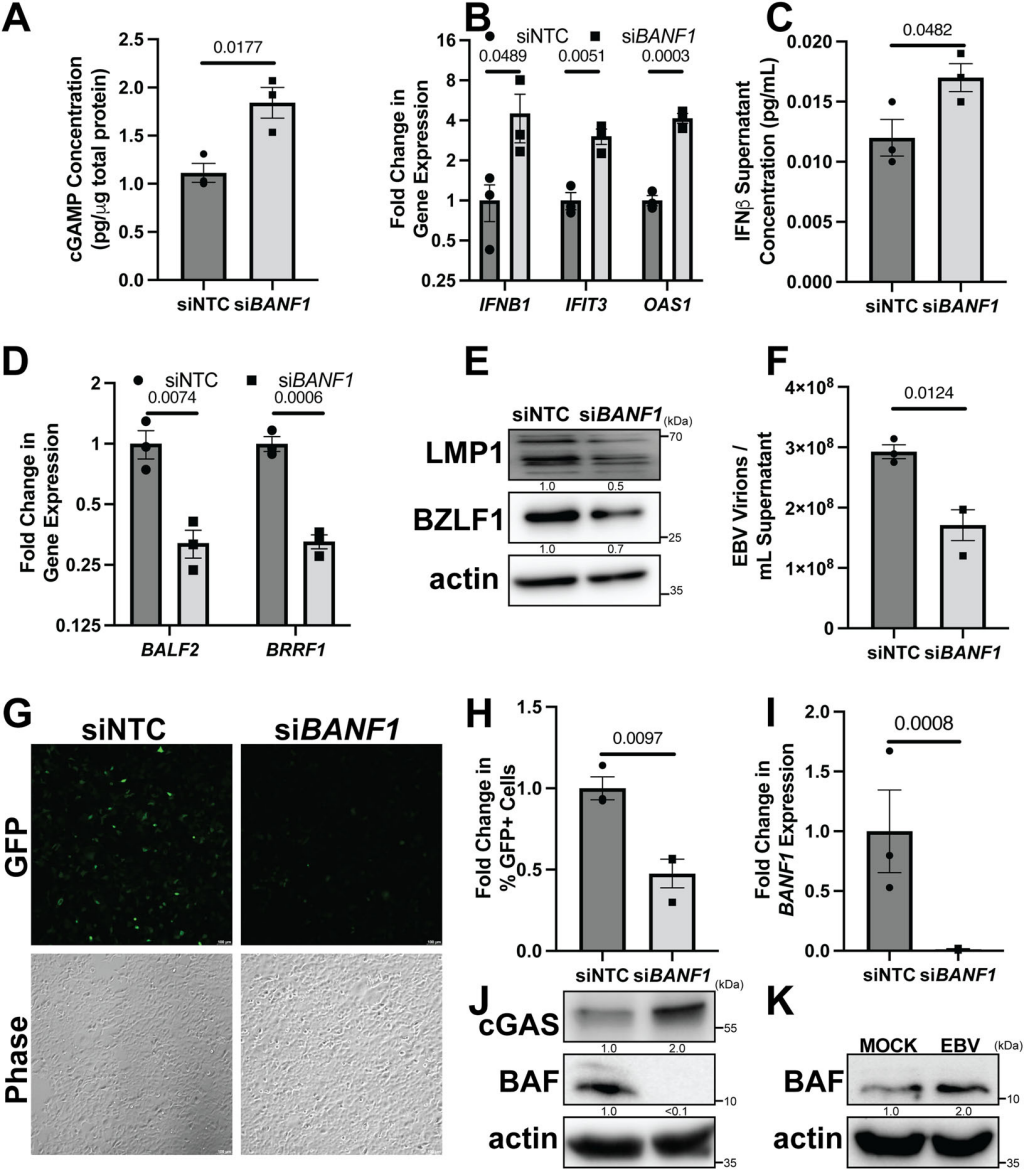
BAF facilitates EBV reactivation from latency in epithelial cells. AGS-EBV cells were transfected with NTC or *BANF1* targeting siRNA for 48 h prior to the addition of 5 ng/mL TPA. **A** Cell lysates were collected at 72 h post-TPA treatment and analyzed by 2’3’-cGAMP ELISA. **B** Cells were harvested and RNA was isolated at 48 h post-TPA treatment. RT-qPCR was subsequently performed to determine ISG mRNA expression levels. **C** Culture supernatant was harvested at 72 h post-TPA treatment and analyzed by IFNβ ELISA. **D** Cells were harvested and RNA was isolated at 72 h post-TPA treatment and RT-qPCR was performed to quantify viral mRNA transcripts. **E** Cell lysates were prepared at 72 h post-TPA treatment and analyzed by western blotting with the indicated antibodies. **F** Culture supernatants were harvested at 72 h post-TPA treatment and DNase treated prior to DNA extraction. DNase-resistant EBV genomes were quantified by real-time qPCR to assess viral load. **G** At 72 h post-TPA treatment, culture supernatants were used to infect naive HEK293 cells. Forty-eight h post infection, GFP + infected cells were analyzed by fluorescent microscopy. **H** Cells were also quantified by flow cytometry. **I** Cells were harvested for RNA at 48 h post-siRNA transfection and subsequent RT-qPCR was performed to quantify *BANF1* mRNA transcripts. **J** Cell lysates were prepared at 48 h post-siRNA transfection and analyzed by western blotting with the indicated antibody. **K** Naive AGS cells were infected with equivalent units of concentrated cell-free EBV or PBS (mock). Cell lysates were prepared 6 h post-infection and analyzed by western blotting. *P* values are the result of two-tailed Student’s *T* tests unless otherwise specified. Error bars indicate the standard error of the mean of three independent biological replicates. Source data are provided as a source data file.

Since we had established that BAF plays a pro-viral role in KSHV lytic reactivation by suppressing cGAS-STING-IRF3 signaling, we were interested if this finding was generalizable to other gammaherpesviruses, such as EBV. Since EBV is associated with gastric cancers, we used the AGS-EBV cell line, a gastric cancer epithelial cell line latently infected with GFP-expressing recombinant EBV that can be stimulated to lytically reactivate via 12-O-tetradecanoyl phorbol 13-acetate (TPA)-induced protein kinase C (PKC) activation^49,50^. First, after stimulation with TPA, lytically reactivating AGS-EBV cells transfected with *BANF1* siRNA produced significantly more cGAMP than cells transfected with NTC siRNA (Fig. 6A). Second, increased cGAS signaling was correlated with higher cGAS protein expression prior to reactivation (Fig. 6J). Third, increased activation of the type I interferon response in BAF-depleted reactivating AGS-EBV cells was evident from increased ISG (*IFIT3* and *OAS1*) mRNA expression, as well as increased expression of *IFNB1* mRNA, which correlated with increased levels of IFNβ protein in the supernatant (Fig. 6B, C). Fourth, this correlated with lower expression levels of EBV mRNAs (*BALF2* and *BRRF1*) and proteins (LMP1 and BZLF1) in reactivated AGS-EBV cells transfected with *BANF1* siRNA compared to cells transfected with NTC siRNA (Fig. 6D, E). Fifth, restriction of viral gene and protein expression in BAF-depleted and reactivated AGS-EBV cells led to a reduction in encapsidated genomes and infectious potential of the supernatant when transferred to naive HEK293 cells compared to the control (Fig. 6F–H and Supplementary Fig. 6A). BAF depletion in AGS-EBV cells was validated by RT-qPCR for *BANF1* mRNA transcripts and western blotting (Fig. 6I, J). These experiments confirmed a general role for BAF in gammaherpesvirus reactivation. To investigate whether BAF expression changed during EBV primary infection, we infected naive AGS cells with cell-free EBV. Six hours post-infection with cell-free EBV, BAF protein levels were increased compared to mock infection in AGS cells (Fig. 6K), suggesting that co-option of BAF-mediated immune evasion during primary infection is common for both KSHV and EBV. Efficient infection of the AGS cell population was confirmed by fluorescent microscopy for GFP expression and western blotting for Epstein–Barr nuclear antigen (EBNA1) expression (Supplementary Fig. 6B, C).

Since EBV is also an etiologic agent of B-cell lymphomas, we were interested in whether BAF played a pro-viral role in lytic reactivation of EBV-infected B cells as well^51^. To test this hypothesis we used the Burkitt lymphoma cell line Akata-BX1, which latently harbors a recombinant GFP-expressing EBV and whose B-cell receptor can be targeted with anti-human IgG to trigger EBV reactivation^52^. After treatment with anti-human IgG, Akata-BX1 cells transfected with *BANF1* siRNA accumulated significantly higher levels of type I interferon response-related mRNAs, such as *IFNB1* itself and ISGs, *IFIT2* and *OAS2* (Supplementary Fig. 7A). Increased antiviral gene expression in BAF-depleted reactivating Akata-BX1 cells correlated with decreased EBV gene (*BALF2* and *BRRF1*) transcription and protein (LMP1 and BZLF1) expression (Supplementary Fig. 7B, C). Moreover, reactivated Akata-BX1 cells that had been transfected with *BANF1* siRNA produced fewer encapsidated EBV virions and the culture supernatant was less infectious when transferred to naive HEK293 cells compared to supernatant from NTC siRNA-treated cells (Supplementary Fig. 7D–G). BAF knockdown efficiency in Akata-BX1 cells was confirmed by RT-qPCR for *BANF1* mRNA transcripts and western blotting (Supplementary Fig. 7H, I). Altogether, these data suggest that BAF plays a similar pro-viral role in EBV and KSHV lytic reactivation in various physiologically relevant cell types by suppressing type I interferon responses, positioning BAF as an important facilitator of the gammaherpesviral lifecycle.

## Discussion

Evading nucleic acid sensors is important for gammaherpesviruses, which possess large dsDNA genomes that encode many genes and establish latency in the nucleus. Gammaherpesviruses target innate immune signaling using virally- and host-encoded factors, e.g., KSHV evasion of RLR sensing: KSHV ORF64 de-ubiquitinates RIG-I, hampering activation, while host factors e.g. adenosine deaminase acting on RNA 1 (ADAR1) and NOD-like receptor X1 (NLRX1) decrease interferon activation by preventing RLR-stimulatory dsRNA from forming or inhibiting the RLR adapter, mitochondrial antiviral-signaling protein (MAVS)^16,53,54^. Similarly, EBV recruits E3 ligase C-terminus of Hsc70-interacting protein (CHIP) to degrade the RIG-I sensor^55^, while EBV protein BPLF1 mediates ubiquitination of RIG-I cofactor tripartite motif-containing protein 25 (TRIM25) to block pathway activation^56^. For DNA viruses, evading cGAS-STING signaling is also important, as evidenced by the many herpesviral factors that suppress cGAS-STING signaling, such as KSHV KicGAS, EBV BLRF2, and KSHV LANA^34,35^. However, the role of regulatory components of the cGAS-STING pathway in facilitating the gammaherpesviral lifecycle had not yet been well-characterized.

BAF was initially discovered as a host cofactor for the integration of human immunodeficiency virus (HIV)^57^. BAF inhibits vaccinia virus replication through its DNA-binding capability^58^ and BAF depletion was associated with decreased replication of multiple viruses^59,60^. The role of BAF during gammaherpesviral latency and the switch to lytic replication had not been explored. Here, we demonstrate that BAF suppresses type I interferon responses during KSHV and EBV lytic reactivation. We found, in agreement with previously published reports^34,61,62^, that the type I interferon response to KSHV reactivation was limited, likely due to suppression by virally-encoded factors. However, reduced levels of BAF made the type I interferon response to KSHV reactivation more pronounced. BAF acted upstream of the cGAS-STING-IRF3 pathway by promoting cGAS protein decay through the proteasomal pathway, decreasing cGAMP production and downstream signaling. *cGAS* mRNA expression was not significantly different between NTC and *BANF1* siRNA-treated cells, confirming a post-transcriptional role for BAF in regulating cGAS protein levels (Supplementary Fig. 4C). cGAS is found at various subcellular sites, such as the nucleus, cytoplasm, and plasma membrane^30,31^. Herpesviral infections produce immunostimulatory DNA that cGAS can detect, such as DNA fragments in the cytoplasm released via herpesviral capsid proteasomal degradation, stress-induced cytoplasmic mitochondrial DNA, or nuclear replicating viral DNA^29,63,64^. BAF restricts cGAS from detecting nuclear and cytoplasmic DNA sources through competitive DNA binding, which is consistent with our findings of DNA-dependent BAF and cGAS-containing complexes (Supplementary Fig. 5E)^41^.

We further demonstrate that BAF plays a role in downregulating antiviral activity in latently KSHV-infected and uninfected cells. Antiviral gene expression signatures in latently infected and reactivating iSLK.219 with minimal *BANF1* expression have commonalities with those of uninfected *BANF1*-KO microglial cells, involving upregulation of genes encoding IFIT, OAS, MX dynamin-like GTPase (MX), radical S-adenosyl methionine domain containing 2 (RSAD2), and ISG15 proteins^59^. Other genes upregulated during BAF depletion were unique to KSHV-infected cells. *HERC5*, which encodes the ISG15 E3 ubiquitin ligase mediator that antagonizes KSHV reactivation^47^, was upregulated in latently infected and lytically reactivating BAF-depleted iSLK.219 cells. Genes encoding known KSHV reactivation antagonists, such as IFI44 and DDX60^48^, were upregulated in reactivating BAF-depleted iSLK.219. Genes encoding cytokine receptors such as interleukin 7 receptor (IL7R) and IL15RA were also induced in BAF-depleted reactivating iSLK.219 cells, which is intriguing since KSHV-infected monocytes upregulate IL-7 and IL-15 expression^65^. IL-7 and IL-15 induce JAK-STAT signaling in B cells, which are relevant in gammaherpesvirus-associated diseases^66^. We also demonstrated a pro-viral role for immunosuppressive BAF activity in uninfected cells in promoting primary infection with KSHV through its impact on cGAS.

The cGAS-STING pathway is also involved in controlling EBV reactivation. EBV infection in airway epithelial cells suppresses signaling downstream of STING through upregulation of tripartite motif-containing protein 29 (TRIM29), an E3 ubiquitin ligase that ubiquitinates and degrades STING^37^. However, host factors controlling cGAS-STING signaling in B cells have not been well studied. The increased type I interferon-stimulated gene expression in BAF-depleted Akata-BX1 cells with reactivating EBV was less dramatic than in the AGS-EBV epithelial cell model, likely due to previously reported dysfunction in cGAS-STING signaling induced by EBV infection in B cells being only partially reversed by BAF depletion^67^.

Altogether, this study demonstrates that the immunosuppressive effect of BAF on the cGAS-STING-IRF3 signaling axis is important for supporting gammaherpesvirus lytic reactivation, which could make it a valuable therapeutic target. Though most cells in gammaherpesvirus-driven tumors exhibit latency, lytic reactivation of infected cells has been implicated in the pathogenesis of KSHV- and EBV-associated disease^8,68^. Since BAF inhibition increases type I interferon responses in a cGAS-dependent manner in multiple cell types, targeting BAF may be a viable approach for treating viral infections and malignancies.

## Methods

### Biosafety statement

All experiments with cell lines and viral infections were carried out in a Biosafety Level 2 facility under the approval of the Biosafety Office in the Department of Environment, Health and Safety and the Institutional Biosafety Committee at the University of North Carolina at Chapel Hill. The laboratory undergoes annual inspections by the Biosafety Office and all personnel undergo annual training in accordance with applicable regulations.

### Cell culture

HEK293 (ATCC), HeLa (ATCC), and SLK cells were maintained in DMEM (Thermo Fisher) containing 10% FBS (VWR), 1% Pen-Strep (Thermo Fisher), and 1% l-glutamine (Thermo Fisher). 293TT cells (a kind gift from Dr. Cary Moody) were maintained in in DMEM containing 10% FBS (Sigma-Aldrich) and 1% Pen-Strep. iSLK.219 cells were maintained in DMEM containing 10% Tet-free FBS (Clontech), 1% Pen-Strep, 1% l-glutamine, 10 μg/mL puromycin (Corning), 250 μg/mL Geneticin (Thermo Fisher), and 400 μg/mL hygromycin B (Corning). BCBL1-TREx-RTA cells (a kind gift from Dr. Jae Jung) were maintained in RPMI 1640 (Corning) containing 10% Tet-free FBS (Clontech), 1% Pen-Strep, 1% l-glutamine, 1% sodium bicarbonate (Thermo Fisher), 0.05 mM β-mercaptoethanol (Thermo Fisher), and 200 μg/mL hygromycin. AGS-EBV cells (a kind gift from Dr. Lindsey Hutt-Fletcher) were maintained in F-12 media (Thermo Fisher) containing 10% FBS (VWR), 1% Pen-Strep, 1% l-glutamine, and 500 μg/mL Geneticin. Akata-BX1 cells (a kind gift from Dr. Lindsey Hutt-Fletcher) were maintained in RPMI 1640 containing 10% FBS (VWR), 1% Pen-Strep, 1% l-glutamine, 0.05 mM mercaptoethanol, and 500 μg/mL Geneticin. AGS cells (ATCC) were maintained in F-12 media containing 10% FBS (VWR), 1% Pen-Strep, and 1% l-glutamine. All cells were maintained at 37 °C and 5% CO_2_ in a regularly cleaned and decontaminated laboratory incubator.

### Viruses

Cell-free concentrated KSHV (rKSHV.219) and EBV were isolated from latently rKSHV.219-infected iSLK.219 cells or latently EBV-infected Akata-BX1 cells, respectively. Virus production was stimulated by replacing iSLK.219 maintenance media with DMEM containing 10% FBS (Clontech), 1% Pen-Strep, 3 μg/mL doxycycline (Thermo Fisher), and 1 mM sodium butyrate (Sigma-Aldrich) for KSHV, and Akata-BX1 maintenance media with RPMI containing 10% FBS (Clontech), 1% Pen-Strep, 25 µg/mL goat anti-human IgG (Jackson Labs) for EBV. The supernatant was harvested 72 h or 120 h after induction, centrifuged to pellet cell debris, and filtered through a sterile 0.45 μm filter (Millipore Sigma). In total, 30 mL of the filtered supernatant was layered over a 5 mL cushion of 20% sucrose (Sigma-Aldrich), and the virus was pelleted by ultracentrifugation in a SW32Ti rotor (Beckman Coulter) at 114,000 × *g* for 2.5 h at 4 °C. After decanting the supernatant, virus pellets were resuspended in 3 mL PBS (Thermo Fisher).

### siRNA transfection and reactivation

iSLK.219 cells in phenol red-free DMEM (Thermo Fisher) containing 10% Tet-free FBS (Clontech) and 1% l-glutamine or AGS-EBV cells in F-12 media containing 10% FBS (VWR) and 1% l-glutamine were reverse transfected with a total concentration of 100 nM siRNA (Horizon) using Lipofectamine RNAiMax (Thermo Fisher) in Opti-MEM (Thermo Fisher) according to the manufacturer’s protocol. After 48 h, iSLK.219 media was replaced with phenol red-free DMEM (Thermo Fisher) containing 10% Tet-free FBS (Clontech), 1% l-glutamine, and 25 ng/mL doxycycline, while AGS-EBV media was replaced with F-12 media containing 10% FBS (VWR), 1% l-glutamine, and 5 ng/mL TPA (Sigma-Aldrich). Cells and supernatants were harvested at various timepoints post-reactivation for downstream analysis. BCBL1-TREx-RTA and Akata-BX1 cells were transfected with 200 nM siRNA using the Cell Line Nucleofector Kit V (Lonza) according to the manufacturer’s protocol and subsequently plated in RPMI 1640 containing 10% Tet-free FBS (Clontech), 1% l-glutamine, 1% sodium bicarbonate, and 0.05 mM β-mercaptoethanol (siRNA sequences can be found in Supplementary Data 2). Forty-eight hours post-nucleofection, BCBL1-TREx-RTA media was changed to 10% Tet-free FBS (Clontech), 1% l-glutamine, 1% sodium bicarbonate, 0.05 mM β-mercaptoethanol, and 1 ng/mL doxycycline, while Akata-BX1 media was changed to 10% Tet-free FBS (Clontech), 1% l-glutamine, 1% sodium bicarbonate, 0.05 mM β-mercaptoethanol, and 10 μg/mL goat anti-human IgG. Cells and supernatants were harvested at various timepoints post-reactivation for downstream analysis.

### Plasmid construction

For pYNC352-*BANF1*-shRNA construction, 2 μg of pYNC352 was digested with BamHI-HF and MluI-HF (NEB) overnight at 37 °C according to the manufacturer’s protocol and subsequently separated on a 0.8% agarose gel and purified using a gel extraction kit (Qiagen). shRNA duplexes were generated by annealing 100 μM forward and reverse primers in annealing buffer (100 mM NaCl, 10 mM Tris-HCl pH 8, 1 mM EDTA) at 95 °C for 4 min, 70 °C for 10 min, and then allowing the reaction to cool to room temperature over the next 2 h (shRNA target sequences are found in Supplementary Data 3). Duplexes were phosphorylated using T4 polynucleotide kinase (NEB) according to the manufacturer’s protocol. Phosphorylated duplexes and linearized pYNC352 were ligated using T4 DNA ligase (NEB) according to the manufacturer’s protocol overnight at room temperature. DH5α cells (NEB) were transformed with the ligation product, and colonies were screened for intact clones by XhoI digestion (NEB) of isolated plasmids according to the manufacturer’s protocol followed by agarose gel electrophoresis. Intact clones were isolated for each independent shRNA (pYNC352-sh*BANF1*-1 and pYNC352-sh*BANF1*-2). The pCMV6-*BANF1*-HA plasmid was generated by Q5 mutagenesis (NEB) on the pCMV6-Entry *BANF1* Human-Tagged ORF Clone (Origene) to replace the original Myc-DDK tag with HA according to the manufacturer’s protocol. Oligonucleotide sequences used for Q5 mutagenesis are found in Supplementary Data 3.

### Lentivirus generation

293TT cells were plated in DMEM containing 10% FBS (Sigma-Aldrich) 16 h prior to the addition of transfection mixture containing FuGENE HD (Promega) transfection reagent, 8 μg pYNC352-shRNA plasmid NS, sh*BANF1*-1, or sh*BANF1*-2, 6 μg psPAX2, and 2 μg pMD2.G diluted in Opti-MEM according to the manufacturer’s protocol. After 6 h, media was changed to DMEM containing 10% FBS (Sigma-Aldrich) and 1% Pen-Strep, and cells were incubated at 37 °C and 5% CO_2_ for an additional 48 h. Supernatants were harvested, spun at 2000*×g* for 5 min to remove cells and debris, and put through a 0.45 μm filter. Lentivirus was pelleted by ultracentrifugation in a SW32Ti rotor (Beckman Coulter) at 114,000*×g* for 2.5 h at 4 °C. After decanting the supernatant, virus pellets were resuspended in 3 mL DMEM containing 10% FBS (Sigma-Aldrich) and incubated at 4 °C overnight before aliquoting and storage at −80 °C.

### Lentiviral transduction and reactivation

TREx-BCBL1-RTA cells were plated in 12-well plates in 500 μL RPMI 1640 containing 10% Tet-free FBS (Clontech), 1% l-glutamine, and 100 μL lentiviral stock. Eight hours later, the media was changed to 1 mL RPMI 1640 containing 20% Tet-free FBS (Clontech) and 1% l-glutamine. After 40 h, 1 additional mL RPMI 1640 containing 20% Tet-free FBS (Clontech) and 1% L-glutamine was added to the wells. Twenty-four h later, cells were counted and equivalent numbers were re-plated in RPMI 1640 containing 10% Tet-free FBS (Clontech), 1% l-glutamine, and 1000 ng/mL doxycycline. After 24 h of stimulation, the cells were washed and the media was changed to RPMI 1640 containing 10% Tet-free FBS (Clontech) and 1% l-glutamine. Cells and supernatants were harvested 4 days post-lytic reactivation.

### Plasmid transfection and reactivation

iSLK.219 cells in phenol red-free DMEM (Thermo Fisher) containing 10% Tet-free FBS (Clontech) and 1% l-glutamine were plated 24 h prior to the addition of transfection mixture containing 6 µg X-tremeGENE 9 DNA transfection reagent (Sigma) and 2 µg pCMV6-*BANF1*-HA in Opti-MEM. After 48 h, the media was replaced with phenol red-free DMEM (Thermo Fisher) containing 10% Tet-free FBS (Clontech), 1% l-glutamine, and 25 ng/mL doxycycline. Cells and supernatants were harvested at various timepoints post-reactivation for downstream analysis.

### DNase-resistant viral genome assay

Cell supernatants were centrifuged at 2000 × *g* to remove cells and debris and filtered through a 0.45-μm filter (Millipore Sigma). Filtered supernatants were treated with 100 U TURBO DNase (Thermo Fisher) per mL supernatant and supplemented to a final concentration of 1× TURBO DNase Buffer (Thermo Fisher). The DNase reaction was allowed to proceed at 37 °C for 1 h before inactivation with 10 mM EDTA (Corning) at 70 °C for 15 min. DNA was extracted using a DNEasy Blood and Tissue Kit (Qiagen) according to the manufacturer’s protocol. Absolute quantification of genome copy number was accomplished by real-time qPCR amplification of *ORF39* (KSHV) or *BMRF1* (EBV) in SensiFast Lo-Rox SYBR (Bioline) with a final primer concentration of 500 nM on a QuantStudio 6 Flex Real-Time PCR System. Standard curves were created using dilutions of pCDNA4/TO-*ORF39*−2XStrep (a kind gift from Dr. Britt Glaunsinger) or pSG5-*BMRF1* (Addgene). Primers used for qPCR are found in Supplementary Data 3. Viral titers were compared statistically using two-tailed homoscedastic Student’s *T* tests to obtain *P* values.

### Viral supernatant transfer

HEK293 cells were plated in six-well plates in phenol red-free DMEM (Thermo Fisher) containing 10% Tet-free FBS (Clontech) and 1% l-glutamine. Twenty-four hours later, supernatants were harvested from reactivated cell cultures and centrifuged at 2000×*g* to pellet cells and debris. Equivalent volumes of supernatant were transferred to the naive HEK293 cells. The negative control and experimental sample wells were equilibrated with serum-free DMEM to a final volume of 2.5 mL and supplemented with 10 μg/mL polybrene (Sigma-Aldrich) before spinfection at 985*×g* for 1.5 h at 30 °C. Wells were supplemented with 1 mL phenol red-free DMEM containing 10% Tet-free FBS (Clontech) and 1% l-glutamine and incubated overnight at 37 °C and 5% CO_2_ before the media was changed to 2 mL phenol red-free DMEM containing 10% Tet-free FBS (Clontech) and 1% l-glutamine. Forty-eight hours post-spinfection, cells were analyzed for GFP positivity by fluorescent microscopy and subsequent mechanical lifting, collection at 2000×*g*, and resuspension in 1 mL PBS for flow cytometry analysis using a MACS-Quant VYB. GFP + cell populations were gated based on negative control samples using FlowJo. Relative sizes of cell populations were compared statistically using two-tailed homoscedastic Student’s *T* tests to obtain *P* values. Supplementary Fig. 9 exemplifies the gating strategy used.

### KSHV and EBV primary infection

SLK cells were plated in a 24-well plate in phenol red-free DMEM containing 10% Tet-free FBS (Clontech) and 1% l-glutamine and reverse transfected with a total concentration of 100 nM siRNA (Horizon) using Lipofectamine RNAiMax in Opti-MEM according to the manufacturer’s protocol. After 48 h, media was replaced with 500 μL serum-free DMEM and either 0.5 μL concentrated cell-free KSHV or PBS for negative control wells. Aliquots of the same virus preparation were used for each of the three biological replicates. Alternatively, SLK or AGS cells were plated in a 24-well plate in phenol red-free DMEM containing 10% Tet-free FBS (Clontech) and 1% l-glutamine or F-12 media containing 10% FBS (Sigma-Aldrich) and 1% l-glutamine, respectively. After 24 h, media was replaced with 500 µL serum-free DMEM or serum-free F-12 media and either 0.5 μL concentrated cell-free KSHV or 100 μL concentrated cell-free EBV, with equivalent volumes of PBS added to negative control wells. Cells were spinfected at 1420 × *g* for 100 min at 30 °C before the media was changed to 500 μL phenol red-free DMEM containing 10% Tet-free FBS (Clontech) and 1% l-glutamine or F-12 media containing 10% FBS (Sigma-Aldrich) and 1% l-glutamine. Forty-eight hours post-spinfection, cells were analyzed for GFP positivity by fluorescent microscopy and subsequent trypsinization (Thermo Fisher), collection at 2000 ×*g*, and resuspension in 1 mL PBS for flow cytometry analysis using a MACS-Quant VYB. GFP + cell populations were gated based on negative control samples using FlowJo. Relative sizes of cell populations were compared statistically using two-tailed homoscedastic Student’s *T* tests to obtain *P* values. Alternatively, infection was confirmed by fluorescent microscopy for GFP signal 72 h post-spinfection prior to lysis and western blotting for latent gammaherpesviral proteins.

### Fluorescent microscopy

GFP and Phase images were taken of HEK293 cells 48 h post-supernatant transfer and of SLK or AGS cells 48 h or 72 h post-primary infection using a Leica Dmi8-inverted microscope. GFP and RFP images were taken of iSLK.219 cells 72 h or 96 h post-reactivation using a Leica Dmi8-inverted microscope. Plates were subsequently scanned on a ClarioStar Plus (BMG) plate reader for RFP and GFP signal to quantify the RFP/GFP intensity ratio. Intensity ratios were compared statistically using two-tailed homoscedastic Student’s *T* tests to obtain *P* values. Images shown are representative of three fields per triplicate well across three biological replicates.

### Western blotting and ELISA

Cell lysates were prepared in RIPA lysis buffer (50 mM Tris-HCl pH 8, 0.1% SDS, 1% NP40, 150 mM NaCl, 30 mM β-glycerol phosphate, 50 mM NaF, 100 μM Na_3_VO_4_) supplemented with protease and phosphatase inhibitor cocktail (Thermo Fisher) or NP40 lysis buffer (0.1% NP40, 150 mM NaCl, 50 mM Tris-HCl pH 8, 30 mM β-glycerol phosphate, 50 mM NaF, 100 μM Na_3_VO_4_) supplemented with protease and phosphatase inhibitor cocktail. Cell lysates were sonicated and centrifuged at 15,000 × *g* for 10 min at 4 °C to pellet cell debris. The protein concentration of the clarified lysate was calculated using a BCA Assay kit (Thermo Fisher). For coimmunoprecipitation assays, lysates containing 1 mg of protein were treated with 100 U TURBO DNase (Thermo Fisher) per mL lysate (or mock PBS) and supplemented to a final concentration of 1× TURBO DNase Buffer (Thermo Fisher). The DNase reaction was allowed to proceed at 37 °C for 1 h. Lysates were pre-cleared by rotating incubation at 4 °C with 40 μL Protein A/G agarose beads (Santa Cruz) and 2 μL mouse IgG (Fisher Scientific) for 45 min prior to centrifugation at 635×*g* for 5 min. In total, 50 μL of anti-HA agarose antibody beads (Sigma-Aldrich) were washed four times by centrifugation at 635×*g* for 5 min, aspiration of supernatant, and addition of 1 mL RIPA or NP40 lysis buffer. Pre-cleared lysates were added to the washed beads and subjected to rotating incubation at 4 °C overnight prior to centrifugation at 635×*g* for 5 min. The supernatant was removed, and the beads were washed four times by addition of 1 mL NP40 or RIPA lysis buffer, rotating incubation at 4 °C for 5 min, centrifugation at 635×*g* for 5 min, and subsequent removal of the supernatant. Washed beads were treated with 40 μL 2× Laemmli sample buffer (Sigma-Aldrich), heated at 95 °C for 10 min, and centrifuged at 8200×*g* for 30 s prior to collection of the eluted protein. For western blotting, equivalent amounts of protein were separated by SDS-PAGE and subsequently transferred to PVDF membrane (Thermo Fisher). The membrane was blocked with 5% nonfat milk or BSA in TBS-T (Tris-buffered saline containing 0.1% Tween 20) prior to incubation with the primary antibody on a rocker at 4 °C overnight. All antibodies were used at 1:1000 dilution unless otherwise specified. Antibodies used were cGAS (Cell Signaling Technology), Phospho-TBK1/NAK (Ser172) XP (Cell Signaling Technology), TBK1/NAK (Cell Signaling Technology), Phospho-IRF-3 (Ser396) (Cell Signaling Technology), β-actin (used at 1:5000) (Santa Cruz Biotechnology), HA-Tag HRP Conjugate (Cell Signaling Technology), Myc-tag (9B11) mouse mAb (Cell Signaling Technology), KSHV ORF45 (Thermo Fisher Scientific), KSHV K8α (Santa Cruz Biotechnology), BANF1/BAF (Abcam), EBV EBNA1 (Abcam), EBV LMP1 (Abcam), and EBV ZEBRA (Santa Cruz Biotechnology). Western band intensities were quantified for protein stability assays using ImageJ and statistically compared using a nonlinear one-phase decay regression in GraphPad Prism to obtain *P* values. Quantification and blots are included in the source data file. For cGAMP ELISA, clarified lysates were quantified using a 2’,3’-cGAMP ELISA kit (Cayman Chemical) according to the manufacturer’s protocol. cGAMP concentration was calculated by comparison to the standard curve, enabling the calculation of the cGAMP/protein ratio. For IFNβ ELISA, supernatants were concentrated using 3000 kDa molecular weight cutoff centrifugal filters (Millipore Sigma) and subsequently assayed by Human IFN-beta DuoSet ELISA kit (R&D Systems) according to the manufacturer’s protocol. Comparison to the standard curve enabled calculation of the culture supernatant IFNβ level. Molecular concentrations were statistically compared using two-tailed homoscedastic Student’s *T* tests to obtain *P* values.

### RT-qPCR

Cell pellets were extracted for RNA using the RNEasy Plus Mini Kit (Qiagen) according to the manufacturer’s protocol. cDNA was generated from the extracted RNA using the SensiFAST cDNA synthesis kit (Bioline). Quantification of target gene expression was accomplished by qPCR in SensiFast Lo-Rox SYBR (Bioline) with a final primer concentration of 500 nM on a QuantStudio 6 Flex Real-Time PCR System (primer sequences can be found in Supplementary Data 3). Expression levels were normalized to the housekeeping gene *ACTB*, and the data displayed are average fold changes (2^-ΔΔCt^). Gene expression values were statistically compared by two-tailed heteroscedastic Student’s *T* tests on ΔCt values to obtain *P* values. For KSHV genome profiling, cDNA from two biological replicates was submitted to the UNC School of Medicine Vironomics Core for real-time qPCR using the KSHVv2.0 viral array (Dittmer, 2003). Viral gene expression was normalized to the geometric mean of three housekeeping gene controls (*ACTB*, *B2M*, and *GAPDH*), and the data displayed are Z-scores of average fold changes (2^-ΔΔCt^) visualized with Partek Flow. For characterization of type I interferon-stimulated gene expression, RT^2^ First Strand cDNA Synthesis Kit (Qiagen) was used to amplify cDNA from two biological replicates for analysis via the RT^2^ Type I Interferon Response PCR Array (Qiagen) according to the manufacturer’s protocol. ISG expression was normalized to the geometric mean of four housekeeping gene controls (*ACTB*, *B2M*, *GAPDH*, and *HPRT1*), and the data displayed are Z-scores of average fold changes (2^-ΔΔCt^) visualized with Partek Flow.

### RNA-Seq

RNA samples were subjected to RNA sequencing by GeneWiz (AZENTA Life Sciences) using Illumina NovaSeq technology. Differential gene expression analysis was conducted by GeneWiz. Read counts in libraries from different samples were normalized to control for factors such as sequencing yield. Differentially expressed genes were called using DESeq2. *P* values and log2 fold changes were generated using the two-tailed Wald test with adjustments for multiple comparisons and graphically displayed in a volcano plot generated using VolcaNoseR (https://huygens.science.uva.nl/VolcaNoseR/)^69^. Gene ontology analysis was conducted and significant enrichment values were determined using the one-tailed Fisher exact test with adjustments for multiple comparisons, which were then visualized in Prism. The RNA-Seq data are available using GEO accession # GSE200781.

### Statistics and reproducibility

Error bars in figures represent the SEM of at least three biological replicates unless otherwise stated. Statistical significance was defined as *P* < 0.05 using a two-tailed Student’s *T* test in Microsoft Excel. RNA-Seq adjusted *P* values were calculated using the two-tailed Wald test with adjustments for multiple comparisons, and the significance of gene ontology terms was determined using the one-tailed Fisher exact test with adjustments for multiple comparisons. Half-lives of nonlinear fit exponential decay models were calculated and decay constants (K) were statistically compared using the Extra sum-of-squares two-tailed F Test with no adjustment for multiple comparisons in GraphPad Prism. No data were excluded from the analyses. The experiments were not randomized or blinded as all experiments were performed in vitro.

## Supplementary information

Supplementary Information

Description of Additional Supplementary Files

Supplementary Data 1

Supplementary Data 2

Supplementary Data 3

## Data Availability

The RNA-Seq data are available using GEO accession # GSE200781. Interferon-stimulated genes were identified using the publicly available database Interferome (http://www.interferome.org)^70^. Unique plasmids generated for this study are available upon request. All the Source data are provided with this paper.

## Source data

Source Data
