# Supplementary material for: Barrier-to-autointegration factor 1 promotes gammaherpesvirus reactivation from latency
Source: Nat Commun. 2023 Feb 6;14:434. doi: 10.1038/s41467-023-35898-2 (PMC9902469; doi:10.1038/s41467-023-35898-2)
Supplement: Supplementary file 1 — Supplementary Information [file 41467_2023_35898_MOESM1_ESM.docx]

**Supplementary Information**

**Supplementary Figure 1. BAF is Required for Optimal KSHV Reactivation from Latency.** Related to Figure 1. TREx-BCBL1-RTA cells were transfected with non-targeting control (NTC) siRNA or *BANF1* targeting siRNA 48h prior to addition of 1 ng/mL doxycycline. (A) Cells were harvested for RNA at 48h post-doxycycline treatment and subsequent RT-qPCR was performed to quantify expression of viral mRNA transcripts. (B) Cell lysates were prepared at 48h post-doxycycline treatment and analyzed by Western blotting with the antibodies indicated. (C) Cells were harvested and RNA isolated at 48h post-siRNA transfection and RT-qPCR was subsequently performed to quantify *BANF1* mRNA transcripts. (D) Cell lysates were prepared at 48h post-siRNA transfection or and analyzed by Western blotting with the indicated antibody. p-values are the result of two-tailed Student’s T tests unless otherwise specified. Error bars indicate the standard error of the mean of 3 independent biological replicates. Source data are provided as a source data file.

**Supplementary Figure 2. BAF antagonizes the cGAS-STING response to KSHV Reactivation.** Related to Figure 2. TREx-BCBL1-RTA cells were transfected with siRNA for 48h prior to addition of 1 ng/mL doxycycline. (A) Cells were harvested for RNA at 24h post-doxycycline treatment and RT-qPCR was subsequently performed to determine ISG mRNA expression levels. (B) Culture supernatants were harvested at 48h post-doxycycline treatment and DNase treated prior to DNA extraction. DNase-resistant KSHV genomes were quantified by real time qPCR. (C) iSLK.219 culture supernatants were transferred to naïve HEK293 cells at 72h post-doxycycline treatment. GFP+ infected cells were quantified by flow cytometry. p-values are the result of two-tailed Student’s T tests unless otherwise specified. Error bars indicate the standard error of the mean of 3 independent biological replicates. Source data are provided as a source data file.

**Supplementary Figure 3. Increased BAF Expression Promotes KSHV Reactivation.** Related to Figure 3. iSLK.219 cells were transfected with either pCMV6 (EV) or pCMV6-BANF1-HA (BAF) expression plasmid for 48h prior to addition of 25 ng/mL doxycycline. At 96h post-doxycycline treatment, culture supernatants were used to infect naïve HEK293 cells. GFP+ infected cells were measured 48h later by flow cytometry. Source data are provided as a source data file.

**Supplementary Figure 4. BAF Promotes KSHV Reactivation through a cGAS-Dependent Mechanism** Related to Figure 4. iSLK.219 cells were transfected with NTC, *BANF1*, *cGAS*, or *BANF1* and *cGAS* targeting siRNA at a total siRNA concentration of 100 nM for 48h prior to addition of 25 ng/mL doxycycline. (A) Cells were harvested for RNA at 72h post-doxycycline treatment and subsequent RT-qPCR was performed to determine KSHV lytic mRNA expression levels. (B) At 72h post-doxycycline treatment, culture supernatants from iSLK.219 cells were used to infect naïve HEK293 cells. At 48 hours post-transfer, GFP+ infected cells were quantified by flow cytometry. (C) Cells were harvested for RNA at 48h post-siRNA treatment and subsequent RT-qPCR was performed to determine *BANF1* and *cGAS* mRNA expression levels. p-values are the result of two-tailed Student’s T tests unless otherwise specified. Error bars indicate the standard error of the mean of 3 independent biological replicates. Source data are provided as a source data file.

**Supplementary Figure 5. BAF Suppresses the Antiviral State in Uninfected, Latently Infected, and Lytically Infected Cells. (A)** iSLK.219 cells were transfected with *BANF1* or NTC siRNA for 48h prior to addition of 100 μg/mL cycloheximide. Lysates were created at the indicated timepoint. cGAS one-phase exponential decay curves for *BANF1* (B) overexpression or (C) siRNA treatment experiments were constructed and analyzed statistically in GraphPad Prism by comparison of nonlinear regression one phase decay constants (K) with the Extra sum-of-squares two-tailed F Test with no adjustment for multiple comparisons. (D) HeLa cells were transfected with 15 μg each of 2 plasmids expressing HA-tagged BAF (pCMV6-*BANF1*-HA), myc-tagged cGAS (pUNO-*CGAS*-MYC), or corresponding empty vector (EV) controls. Lysates were collected in RIPA buffer 72h post-transfection and subjected to immunoprecipitation with anti-HA agarose beads. Bound proteins were eluted and detected by Western blot. (E) HeLa cells were transfected with 2 plasmids expressing HA-tagged BAF (pCMV6-*BANF1*-HA), myc-tagged cGAS (pUNO-*CGAS*-MYC), or corresponding EV controls. Lysates were collected in NP40 buffer 72h post-transfection and either DNase- or mock-treated prior to immunoprecipitation with anti-HA agarose beads. Bound proteins were eluted and detected by Western blot. Immunoprecipitation results are representative of 3 independent biological replicates. (F-G) Naïve SLK cells were infected with equivalent units of concentrated cell-free KSHV or PBS (mock). Infection efficiency was assayed via LANA expression by Western blot in panel F or GFP expression by fluorescent microscopy 72h post-infection in panel G. (H) Naïve SLK cells were transfected with NTC siRNA or *BANF1* targeting siRNA for 48h prior to infection with equivalent units of concentrated cell-free KSHV. GFP+ infected cells were analyzed at 48h post-infection by flow cytometry. Primary infection data are representative of 3 independent biological replicates. Source data are provided as a source data file.

**Supplementary Figure 6. BAF Facilitates EBV Reactivation from Latency in Epithelial Cells.** Related to Figure 6. (A) AGS-EBV cells were transfected with NTC or *BANF1* targeting siRNA for 48h prior to addition of 5 ng/mL TPA. At 72h post-TPA treatment, culture supernatants were used to infect naïve HEK293 cells. At 48 hours post-infection, GFP+ infected cells were analyzed by flow cytometry. Naïve AGS cells were infected with equivalent units of concentrated cell-free EBV or PBS (mock). 72h post-infection, cells were (B) analyzed for GFP signal by fluorescent microscopy and (C) lysates were created and analyzed by Western blotting. Primary infection data are representative of 3 independent replicates. Source data are provided as a source data file.

**Supplementary Figure 7. BAF Facilitates EBV Reactivation from Latency in B Cells. BAF Facilitates EBV Reactivation from Latency in B Cells.** Akata-BX1 cells were transfected with NTC or *BANF1* targeting siRNA for 48h prior to addition of 10 µg/mL anti-human IgG. (A) Cells were harvested at 96h post-anti-IgG treatment and RNA was isolated. RT-qPCR was performed to determine ISG mRNA transcript levels. (B) Cells were harvested at 120h post-anti-IgG treatment and RNA was isolated. RT-qPCR was performed to quantify expression of viral mRNA transcripts. (C) Cell lysates were prepared at 120h post-anti-IgG treatment and analyzed by Western blotting with the indicated antibodies. (D) Culture supernatants were harvested at 120h post-anti-IgG treatment and DNase treated prior to DNA extraction. DNase-resistant EBV genomes were quantified by real time qPCR to determine viral loads. (E) At 120h post-anti-IgG treatment of Akata-BX1 cells, culture supernatants were transferred to naïve HEK293 cells. At 48 hours post-transfer, GFP+ infected cells were quantified by (G) flow cytometry and (F) analyzed by fluorescent microscopy. (H) Cells were harvested at 48h post-siRNA transfection and RNA was isolated. RT-qPCR was performed to quantify *BANF1* mRNA transcript levels. (I) Cell lysates were prepared at 48h post-siRNA transfection and analyzed by Western blotting with the indicated antibody. p-values are the result of two-tailed Student’s T tests unless otherwise specified. Error bars indicate the standard error of the mean of 3 independent biological replicates. Source data are provided as a source data file.

**Supplementary Figure 8. Comparison across reactivation contexts.** Results from (A) cGAMP ELISA, (B) IFNβ ELISA, (C) KSHV genome quantitation, and (D) EBV genome quantitation experiments were visualized on a common axis to enable comparisons of relative inductions of cellular responses to lytic reactivation under various contexts. Please note that different cell types are used and each cell type has a different basal level of interferon. p-values are the result of two-tailed Student’s T tests unless otherwise specified. Error bars indicate the standard error of the mean of 3 independent biological replicates. Source data are provided as a source data file.

**
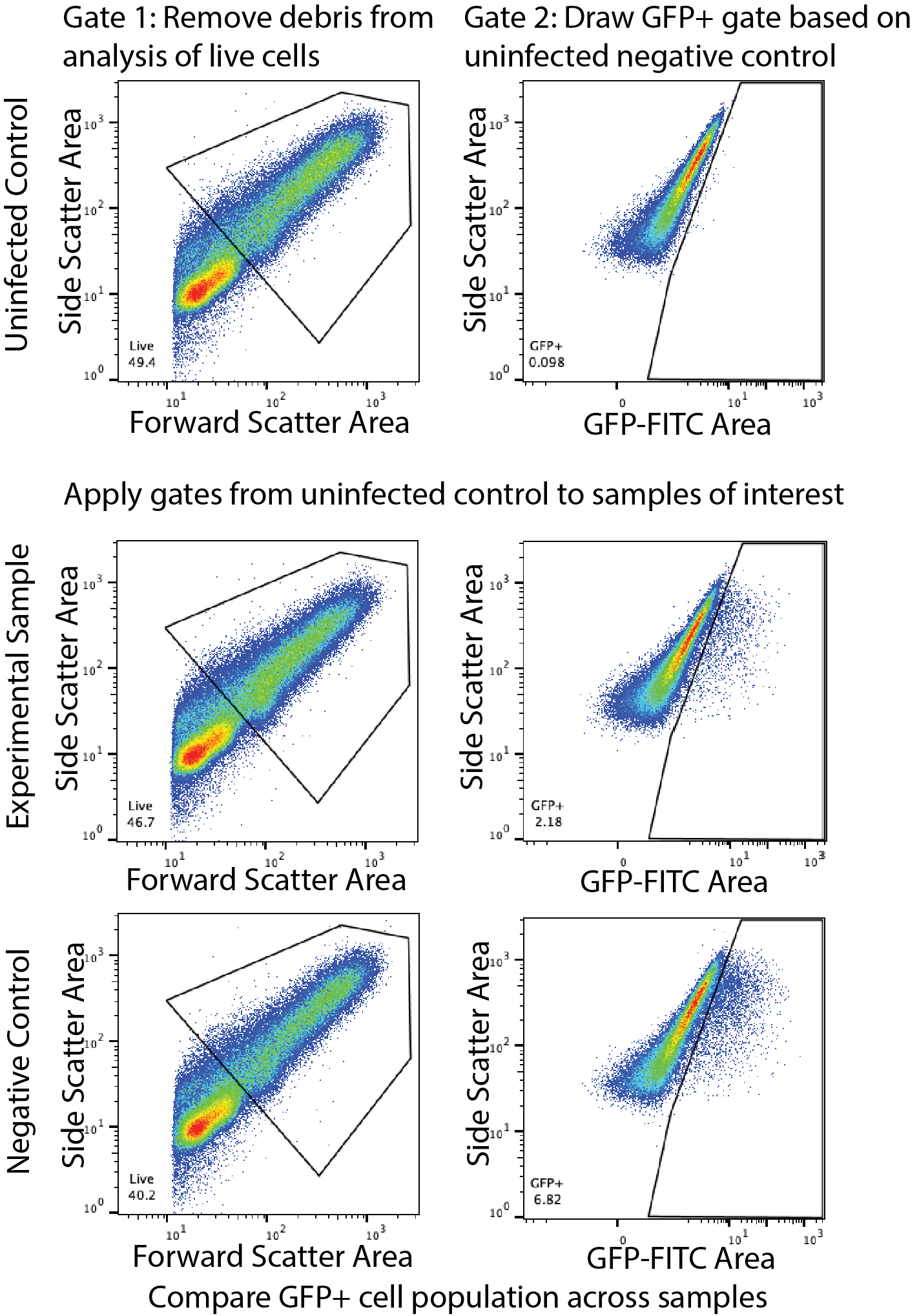
**

**Supplementary Figure 9. Flow Cytometry Gating Strategy.** A visual demonstration of the process used to identify the percentage of cells belonging to the GFP+ population in various experiments throughout the manuscript.

**Supplementary Data 1. Genes with Significantly Altered Expression after *BANF1* Knockdown.** iSLK.219 cells were transfected with NTC siRNA or *BANF1* targeting siRNA for 48h prior to addition of 25 ng/mL doxycycline. Cells from two biological replicates were harvested for RNA at 0h and at 48h post-doxycycline treatment and subjected to RNA-Seq analysis. This table contains the fold changes and p-values (as determined by two-tailed Wald test with adjustment for multiple comparisons) for each gene with significantly altered expression between NTC and *BANF1* siRNA treatment.

**Supplementary Data 2. siRNA Sequences Used for Alteration of Gene Expression.** This table contains the pooled siRNA sequences used to target expression of the *BANF1* and *cGAS* genes, as well as the non-targeting control (NTC) sequences.

**Supplementary Data 3. Oligonucleotide Sequences Used for shRNA Plasmid Construction, RT-qPCR, Q5 Mutagenesis, and Viral Genome Quantitation.** This table contains the oligonucleotide sequences used to generate shRNA lentiviral plasmids targeting *BANF1* expression, quantify gene expression via RT-qPCR, generate the HA-tagged *BANF1* expression plasmid, and quantify KSHV and EBV viral genomes in the supernatant of reactivating cell cultures via qPCR.
