# Supplementary material for: Barrier-to-autointegration factor 1 promotes gammaherpesvirus reactivation from latency
Source: Nat Commun. 2023 Feb 6;14:434. doi: 10.1038/s41467-023-35898-2 (PMC9902469; doi:10.1038/s41467-023-35898-2)
Supplement: Supplementary file 2 — Description of Additional Supplementary Files [file 41467_2023_35898_MOESM2_ESM.pdf]

Description of Additional Supplementary Files

File Name: Supplementary Data 1
Description: **Genes with Significantly Altered Expression after BANF1 Knockdown.** iSLK.219 cells were transfected with NTC siRNA or BANF1 targeting siRNA for 48h prior to addition of 25 ng/mL doxycycline. Cells from two biological replicates were harvested for RNA at 0h and at 48h post-doxycycline treatment and subjected to RNA-Seq analysis. This table contains the fold changes and p-values (as determined by two-tailed Wald test with adjustment for multiple comparisons) for each gene with significantly altered expression between NTC and BANF1 siRNA treatment.

File Name: Supplementary Data 2
Description: **siRNA Sequences Used for Alteration of Gene Expression.** This table contains the pooled siRNA sequences used to target expression of the BANF1 and cGAS genes, as well as the non-targeting control (NTC) sequences.

File Name: Supplementary Data 3
Description: **Oligonucleotide Sequences Used for shRNA Plasmid Construction, RT-qPCR, Q5 Mutagenesis, and Viral Genome Quantitation.** This table contains the oligonucleotide sequences used to generate shRNA lentiviral plasmids targeting BANF1 expression, quantify gene expression via RT-qPCR, generate the HA-tagged BANF1 expression plasmid, and quantify KSHV and EBV viral genomes in the supernatant of reactivating cell cultures via qPCR.
